# Associations Between C-Reactive Protein Levels, Exercise Addiction, and Athlete Burnout in Endurance Athletes

**DOI:** 10.3389/fpsyg.2021.615715

**Published:** 2021-06-04

**Authors:** Linda Becker, Anna Dupke, Nicolas Rohleder

**Affiliations:** Chair of Health Psychology, Department of Psychology, Friedrich-Alexander-Universität Erlangen-Nürnberg, Erlangen, Germany

**Keywords:** exercise addiction, athlete burnout, C-reactive protein, inflammation, endurance athletes, physical activity, health behavior

## Abstract

Regular physical activity can support long-term health maintenance, e.g., by reducing inflammatory markers such as C-reactive protein (CRP) levels. However, excessive physical activity can promote the development of both mental and physical illness as well. From a psychological perspective, excessive exercise can lead to the development of exercise addiction (EA) and athlete burnout (AB). However, EA and AB have been rarely investigated so far and it is still unknown whether they are associated with risk factors for physical diseases such as increased CRP levels. In our study, we investigated whether EA and AB in endurance athletes are associated with CRP concentrations. Furthermore, sex differences and prevalence rates of EA were investigated. Ninety-five endurance athletes participated (54.7% female, mean age = 31.8 ± 15.02 years). CRP levels were assessed by means of Dried Blood Spots. For EA and AB assessment, the Exercise Addiction Inventory (EAI) and the Athlete Burnout Questionnaire (ABQ) were used. Exercise addiction was negatively associated with CRP in men. No associations were found for women. None of the ABQ subscales (physical and emotional exhaustion, devaluation of sports, and reduced sense of accomplishment) was associated with CRP levels or with EA. Prevalence of EA was 4.2%. More than 80% of the participants were at risk for EA development. Our results suggest that EA is not necessarily associated with a higher risk for physical diseases through inflammatory pathways. However, EA is a serious mental illness that is widespread in athletes, at least at a subclinical level.

## Introduction

Regular physical activity (PA) usually has a positive connotation and is widely associated with promoting mental and physical health (Alfermann and Stoll, 1997; Graf et al., 2004; Stoll and Ziemainz, 2012). One important potential health benefit of regular PA is that it can contribute to a reduction of inflammatory processes (Ford, 2002; Bruunsgaard, 2005) and can, therefore, promote physical health in the long-term (Roubenoff, 2007). However, in recent years another side of PA has also come into focus, namely the negative aspects of too much exercising. From a psychological perspective, excessive exercising can lead to the development of exercise addiction (EA; Stoll, 2017). Exercise addiction belongs to the non-substance-related addictions and has not yet been part of common classification systems. Divergent prevalence rates for EA have been reported for different groups [e.g., 1–3% (Stoll, 2017), 4.5% (Ziemainz et al., 2013), 5% (Lichtenstein and Jensen, 2016), or 30.7% (Trott et al., 2020)]. Exercise addiction is—just like other addictions—accompanied by withdrawal symptoms if too long breaks are taken, tolerance development, and loss of control (Breuer and Kleinert, 2009; Ziemainz et al., 2013; Stoll, 2017). Regardless of competition ambitions, there is an inner constraint to do sports. Currently, it is mainly discussed whether EA should be classified as an addiction in the sense of a substance-independent dependency, an obsessive-compulsive disorder, or an impulse control disorder. Little is known whether and how EA is related to other diseases and inflammatory processes. Other addictions, especially substance dependencies, have been found to be related with an increase in inflammatory markers such as C-reactive protein (CRP; Reece, 2012; Costello et al., 2013). However, the associations between substance-independent addictions (such as EA) and CRP levels are not yet fully understood.

Another psychological risk that is associated with excessive exercising, is athlete burnout (AB). Athlete burnout symptoms are closely related to burnout conditions that have been described in the working context. Burnout in general is accompanied by the core symptoms emotional exhaustion, depersonalization, and reduced performance (Maslach and Jackson, 1986). In the exercise context, the burnout concept has been adapted. Three dimensions of AB are usually reported (Raedeke et al., 2002): physical and emotional exhaustion (E), devaluation (D) of sports, and reduced sense of accomplishment (RA). The first dimension, E, represents a consequence of the intense training sessions and competitions. The second dimension, D, is often associated with a loss of interest and a cynical attitude toward one’s own sports. The third dimension, RA, refers to the negative evaluation of one’s own abilities, achievements, and successes and occurs when the athletes are unable to achieve their personal goals or remain below their own expectations (Raedeke et al., 2002; Gustafsson et al., 2017). As in the organizational context, the core symptom of AB is the E dimension, which has been described as the extreme end-stage of maladjustment to the training or to insufficient recovery (Goodger et al., 2007; Gustafsson et al., 2016). The associations between burnout and CRP levels that have been reported so far are divergent: For example, Toker et al. (2005) reported increased CRP levels in women with high levels of burnout symptoms, but no associations for men. In contrast, Grossi et al. (2003) failed to find any association between CRP and burnout in women. This has been supported by a meta-analysis by Danhof-Pont et al. (2011), who also did not find an association between CRP levels and burnout. The physiological health consequences of AB, especially the associations with inflammation, have been investigated only little so far. Therefore, it is still unknown if AB symptoms are associated with inflammatory processes (e.g., CRP levels) or not.

The associations between AB and EA have also been rarely investigated so far. Reche et al. (2018) found a positive correlation between EA and both the E and D dimensions of AB as well as a marginally negative correlation between EA and the RA dimension of AB. In the working context, a positive association between work addiction and burnout has been reported (Schaufeli et al., 2008).

Overall, more research is needed in which the associations between EA, the sub-dimensions of AB, and inflammation are investigated. The aim of our study was to contribute new knowledge to this research gap. We used an ad-hoc sample of endurance athletes with the aim to recruit a sub-sample of participants with severe levels of EA and AB as well as a sub-sample with sub-clinical, or without any EA or AB symptoms. Furthermore, we aimed to investigate potential sex differences and to estimate prevalence rates of EA in this group of endurance athletes.

## Materials and Methods

### Participants

Participants were *N* = 95 German-speaking endurance athletes (54.7% female, mean age = 31.8 ± 15.02 years, min: 18, max: 74 years, body-mass index (BMI) = 22.5 ± 2.56 kg/m^2^, min: 16.2, max: 28.7 kg/m^2^). All participants gave their written and informed consent. Data was anonymized directly after collection to protect participant’s privacy. The study was conducted according to the principles expressed in the Declaration of Helsinki and was approved by the local ethics committee of the Friedrich-Alexander University Erlangen-Nürnberg (FAU, # 194_18 B). A power calculation yielded a minimum sample size of *N* = 82 participants (correlational analysis, two-tailed, medium effect size *r* = 0.30, α = 0.05, 1–β = 0.80).

### Questionnaires

Exercise addiction was assessed by means of a German version of the Exercise Addiction Inventory (EAI; Terry et al., 2004; Ziemainz et al., 2013). The EAI is a self-report questionnaire that assesses the degree of risk to develop exercise addiction regardless of the type of sports. The EAI includes six items that should be answered on a 5-point Likert scale from 0 to 4. For evaluation, the item values are summed. According to the recommendations of Terry et al. (2004), the following categories can be assigned: no symptoms of EA (0–12), risk for EA (13–23), and EA (≥24).

For AB assessment, a German Version of the Athlete Burnout Questionnaire (ABQ; Raedeke et al., 2002; Ziemainz et al., 2004) was used. The ABQ assesses the three AB dimensions E, D, and RA within three subscales. No categorization was conducted because no cut-off values were available.

The amount of regular PA was assessed by means of the “Bewegungs- und Sportaktivität Fragebogen” (exercise and sport activity questionnaire) (BSA-F; Fuchs et al., 2015). The BSA-F is based on the so-called Frequency-Intensity-Time-and-Type-Technology (Sallis and Owen, 1998), which means that PA is examined by considering the dimensions frequency, duration, intensity, and sports type. For this study, the total amount of PA per week was used, which includes PA during sports as well in leisure time.

### C-Reactive Protein Assessment and Analysis

C-reactive protein levels were assessed by means of Dried Blood Spots (DBS; McDade et al., 2004). The procedure is described in detail elsewhere (e.g., McDade et al., 2007; Danese et al., 2011; McDade, 2014). In short, one of the participant’s fingertips was sprayed with an alcoholic skin antiseptic and wiped with a sterile cellulose swab. A disposable lancet was attached to the side of the disinfected fingertip and released. The first blood drop was wiped off with a cellulose swab and discarded. Approximately 50 μl of capillary blood were then applied to four marked sectors of a special filter paper (Whatman 903, GE Healthcare Life Sciences, Germany). The filter paper was then dried at room temperature for at least 8 h and afterward stored in an airtight envelope at -30°C. For quantification, a circle with a diameter of 3.5 mm was punched out. A “Human C-Reactive Protein/CRP Quantikine ELISA Kit” (R&D Systems) was used for analysis. For determination of absolute CRP serum concentrations from concentrations in DBS, a linear transformation according the formula provided by McDade et al. (2004) was used (Serum CRP = 1.15 DBS-CRP + 0.13).

### General Procedure

Participants were recruited via flyers and announcements in a local sports club and during a local running competition. The inclusion criterion was participation in regular endurance sports. Exclusion criteria were usage of any medication and any diseases that might be associated with inflammatory processes. For CRP collection, appointments between the participants and the experimenter were scheduled. Questionnaires (EAI, ABQ, and BSA-F) were filled out online within the following 3 days after CRP collection. The study was conducted between June 2018 and November 2019.

### Data Analysis

For statistical analyses, IBM SPSS Statistics (version 26) was used. Because of positive skewness, CRP levels and regular PA scores were transformed by means of the natural logarithm (ln) prior to statistical analysis. Partial correlation *r*_part (age, sex, BMI)_ with age, sex, and BMI as covariates were calculated between ln-transformed CRP levels, the EAI score, the scores of the ABQ subscales, and the ln-transformed amount of regular PA. To control for multiple comparisons, an adjusted α-level of α_adjusted_ = 0.05/6 = 0.008 was used for these analyses. For analyzing potential sex differences, partial correlations *r*_part (age, BMI)_ with age and BMI as covariates were calculated between the EA and CRP levels, separately for men and women. For these analyses, an adjusted α-level of α_adjusted_ = 0.05/2 = 0.025 was used. Furthermore, group differences in CRP levels between the three EAI groups were investigated using an analysis of variance (ANOVAs) with the between-subjects group factor EAI group. This ANOVA was conducted for the whole sample only because of too low group sizes when differentiating between men and women.

## Results

### Descriptive Statistics and Prevalence of Exercise Addiction

Overview about the sample’s descriptive statistics and the athlete’s training histories are provided in Tables 1, 2. Four (4.2%, 2 male, 2 female) of the participants were classified as exercise addicted, who met the criterion of an EAI score ≥24. Seventy-seven participants (81.1%, 32 male, 45 female) were at risk for developing EA with EAI scores between 13 and 23, and 14 participants (14.7%, 9 male, 5 female) did not show any EAI symptoms.

**TABLE 1:** Descriptive sample statistics.

	***N***	**Min.**	**Max.**	**Mean**	**Standard deviation**
CRP (mg/l)	95	0.2	6.02	1.30	1.04
ln(CRP)	95	−1.62	1.8	0.02	0.68
BMI (kg/m^2^)	95	16.2	28.7	22.48	2.56
EAI	95	1.33	4.17	2.74	0.62
ABQ-E	95	0.8	3.8	1.84	0.68
ABQ-D	95	1	3.8	2.04	0.70
ABQ-RA	95	1	4.4	2.43	0.76
PA (min/week)	95	49	5,459	728.1	804.1

*CRP, C-reactive protein; ln, natural logarithm; BMI, body-mass index; EAI, Exercise Addiction Inventory; ABQ, Athlete Burnout Questionnaire; E, exhaustion; D, devaluation; RA, reduced accomplishment; PA, physical activity.*

**TABLE 2:** Demographic data and athlete’s training history.

		**Percentage**
Relationship status	Single	37.9
	Married	29.5
	Parnership	25.3
	Separated	3.2
	Divorced	4.2
Highest educational degree	Certificate of secondary education	4.2
	Secondary school level	9.5
	Vocational diploma	3.2
	General qualification for university entrance	47.4
	B.Sc.	13.7
	M.Sc. or diploma	20
	Ph.D.	2.1
Employment status	Student	55.8
	Housewife	2.1
	Employee	27.4
	Self-employed	5.3
	Official	1.1
	On leave	1.1
	Retirement	4.2
	Other	3.2
Competitive sports	Yes	14.7
	No	85.3
Trained by a trainer	Yes	40
	No	60
Number of trainings per week	1	4.2
	2	25.3
	3	20
	4	17.9
	5	15.8
	6	11.6
	7	4.2
	10	1.1
Days without training per week	0	3.2
	1	17.9
	2	23.2
	3	25.3
	4	21.1
	5	8.4
	6	1.1

### Associations Between C-Reactive Protein Levels, Exercise Addiction, and Athlete Burnout

**FIGURE 1:**
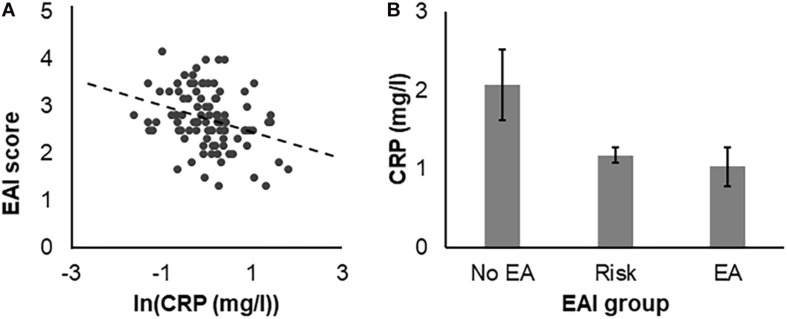
**(A)** Association between C-reactive protein (CRP) levels and exercise addiction (EA) that was assessed by means of the Exercise Addiction Inventory (EAI). **(B)** Mean CRP levels for the three EA groups No EA (*N* = 14), Risk for EA (*N* = 77), and EA (*N* = 4).

A negative association between exercise addiction and CRP levels was found [*r*_part (age, sex, BMI)_ = -0.32, *p* = 0.002]. Participants with higher EAI scores had lower CRP levels and, thus, lower inflammation (Figure 1A). Furthermore, CRP levels were significantly different between the three EAI groups [*F*_(__2, 92)_ = 3.72, *p* = 0.028; Figure 1B]. After correction for multiple comparisons (α_adjusted_ = 0.008) no association between any of the ABQ subscales and CRP levels were found. The amount of regular PA was also not associated with any of the ABQ subscales or with EA. The whole results of the correlational analysis are provided in Table 3.

**TABLE 3:** Results of the correlational analysis for the whole sample (*N* = 95).

		**ln[CRP(mg/l)]**	**EAI**	**ABQ-E**	**ABQ-D**	**ABQ-RA**
EAI	*r*	**−0.317**	1			
	*p*	0.002**				
	*df*	90				
ABQ-E	*r*	0.047	0.141	1		
	*p*	0.656	0.181			
	*df*	90	90			
ABQ-D	*r*	**0.207**	**−0.234**	0.267	1	
	*p*	0.047*	0.025*	0.01		
	*df*	90	90	90		
ABQ-RA	*r*	0.098	−0.096	**0.206**	**0.385**	1
	*p*	0.354	0.362	0.048*	< 0.001***	
	*df*	90	90	90	90	
ln [PA (min/week)]	*r*	−0.028	**0.247**	**0.224**	0.022	0.085
	*p*	0.793	0.018*	0.032*	0.831	0.419
	*df*	90	90	90	90	90

*Partial correlations with age, sex, and body-mass index (BMI) as covariates were calculated (mean age = 31.8 ± 15.02 years, 54.7% female, mean BMI = 22.5 ± 2.56). Significant correlations (p < 0.05) are highlighted in bold.*

CRP, C-reactive protein; ln, natural logarithm; EAI, Exercise Addiction Inventory; ABQ, Athlete Burnout Questionnaire; E, exhaustion; D, devaluation; RA, reduced accomplishment; PA, physical activity.

*p < 0.05, **p < 0.01, and ***p < 0.001.

### Sex Differences

For female athletes, no association between CRP levels and EA was found (*p* > 0.025). For men, EAI scores were negatively related with CRP levels [*r*_part (age, BMI)_ = -0.35, *p* = 0.024].

## Discussion

The aim of the present study was to investigate the associations between CRP levels, EA, and AB in endurance athletes. Furthermore, prevalence rates and sex differences were investigated. Overall, a negative association between CRP levels and EA was found. However, further analyses indicated that this was significant for men only. None of the ABQ subscales was related with any of the other variables (i.e., CRP levels, EA, and PA). Prevalence rate for EA was 4.2%. Furthermore, 81.1% of the athletes were at risk for EA development.

Our result that lower CRP levels were found in athletes with EA than in athletes without any EA symptoms was unexpected because the opposite pattern has been reported in previous studies for other addictions (i.e., higher CRP levels in substance-addicted participants than in healthy controls; Reece, 2012; Costello et al., 2013). However, the findings of these studies do not necessarily have to be comparable to findings for substance-independent addictions such as EA. For substance abuse, the mechanism behind the association between addiction and inflammation is probably that the substances damage the body, which promotes an increase in inflammatory markers. For substance-independent EA, inflammation might be reduced because of the regular PA, in the sense of PA being a health-promoting behavior (e.g., Ford, 2002). Furthermore, EA might be accompanied by further health-promoting behaviors (e.g., eating a healthy diet, weight control) to ensure long-term physical fitness and, thus, sports performance, promoting further reduction of inflammatory markers. Interestingly, the association between EA and CRP levels was found in male athletes only. Sex differences in inflammatory processes have been reported previously (e.g., Thorand et al., 2006; Yang et al., 2013), but the underlying mechanisms are not yet fully understood. Therefore, further research is needed to investigate sex differences in inflammatory processes in athletes.

However, it should be noted that only four participants could be classified as exercise addicted in our sample. Therefore, further research is needed in which larger samples with EA will be compared with an equal number of healthy, non-addicted controls. Our prevalence rate of 4.2% is comparable with the numbers that have been reported by Lichtenstein and Jensen (2016) for CrossFit athletes and by Ziemainz et al. (2013) for endurance athletes, but it is much lower than the number of about 30.7% that has been reported by Trott et al. (2020). However, the number of participants who were at risk for EA development was >80% in our study. This shows the importance of considering EA development as a serious potential psychological health problem.

Another aim of our study was to investigate whether EA is associated with AB. However, we failed to find any association between these constructs. This is contrary to the findings by Reche et al. (2018), who found associations between EA and all AB dimensions (negative direction for RA, and positive for the E and D dimensions) as well as findings from the organizational context (e.g., Schaufeli et al., 2008). Furthermore, we did not find an association between AB and CRP levels. This is in line with findings the organizational context, in which also no associations between burnout and CRP have been found (e.g., Danhof-Pont et al., 2011).

However, further research is needed in which the associations between EA, AB, and inflammation should be investigated. Future research should focus on the underlying psychological (i.e., comorbid mental disorders) and physiological mechanisms (e.g., sex hormones). Furthermore, different sports should be compared.

## Conclusion

We conclude that EA is not necessarily a risk factor for physical diseases through inflammatory pathways. However, it is a serious mental disease that is widespread in athletes, at least at a subclinical level. More research is needed to fully understand the associations between inflammation, EA, and AB as well as their associations with mental and physical health or illness.

## Data Availability Statement

The datasets generated for this study are available on request to the corresponding author.

## Ethics Statement

The studies involving human participants were reviewed and approved by Ethikkommission der FAU Erlangen-Nürnberg. The patients/participants provided their written informed consent to participate in this study.

## Author Contributions

LB and AD planned and conceptualized the study, collected data, and wrote the manuscript. LB and AD analyzed the data and discussed the results with NR. LB and NR supervised the study. All authors reviewed the manuscript and approved the final manuscript.

## Conflict of Interest

The authors declare that the research was conducted in the absence of any commercial or financial relationships that could be construed as a potential conflict of interest.
